# Improvement of a Clinical Score for Necrotizing Fasciitis: ‘Pain Out of Proportion’ and High CRP Levels Aid the Diagnosis

**DOI:** 10.1371/journal.pone.0132775

**Published:** 2015-07-21

**Authors:** Thomas Borschitz, Svenja Schlicht, Ekkehard Siegel, Eric Hanke, Esther von Stebut

**Affiliations:** 1 Center for Coloproctology/Surgery, Mainz, Germany; 2 Department of Dermatology, University Medical Center, Johannes Gutenberg University Mainz, Mainz, Germany; 3 Institute for Medical Microbiology, University Medical Center, Johannes Gutenberg University Mainz, Mainz, Germany; 4 Department of Traumatology/Orthopedics, University Medical Center, Johannes Gutenberg University Mainz, Mainz, Germany; University of Florida, UNITED STATES

## Abstract

Necrotizing fasciitis (NF) is a rare mono-/polymicrobial skin infection that spreads to underlying tissues. NF is quickly progressing and leads to life threatening situations. Immediate surgical debridement together with i.v. antibiotic administration is required to avoid fatal outcome. Early diagnosis is often delayed due to underestimation or confusion with cellulitis. We now compared the initial clinical and laboratory presentation of NF and cellulitis in detail to assess if a typical pattern can be identified that aids timely diagnosis of NF and avoidance of fatal outcome. 138 different clinical and laboratory features of 29 NF patients were compared to those of 59 age- and gender matched patients with severe erysipelas requiring a subsequent hospitalization time of ≥10 days. Differences in clinical presentation were not obvious; however, NF patients suffered significantly more often from strong pain. NF patients exhibited dramatically elevated CRP levels (5-fold, p>0.001). The overall laboratory risk indicator for necrotizing fasciitis (LRINEC) score was significantly higher in NF patients as compared to cellulitis. However, a modification of the score (alteration of laboratory parameters, addition of clinical parameters) led to a clear improvement of the score with a higher positive predictive value without losing specificity. In summary, clinical differentiation of NF from cellulitis appears to be hard. ‘Pain out of proportion’ may be an early sign for NF. An improvement of the LRINEC score emphasizing only relevant laboratory and clinical findings as suggested may aid the early diagnosis of NF in the future leading to improvement of disease outcome by enabling rapid adequate therapy.

## Introduction

Necrotizing fasciitis (NF) is a rare infection of the skin and subcutaneous tissue that easily spreads across the fascial planes [1,2]. The estimated incidence is 0.4/100,000 individuals [3]. Type I NF describes polymicrobial infections, whereas type II NF has a monomicrobial pathogenesis. Many types of bacteria can cause NF, among which group A Streptococci are most common. However, (methicillin-resistant) *Stapylocyoccus aureus* (MRSA), *Clostridium perfringens*, *Bacteriodies fragiles* as well as Gram-negative bacteria have also been detected [4].

Overall, it is well accepted that patients with a compromised immune system are more likely to develop NF. Minor trauma can lead to inoculation of the pathogen. The pathogenesis is in part dependent on the release of toxins by the bacteria (e.g. streptococcal pyogenic exotoxin, or superantigens), which are known to activate T cells in a non-specific manner leading to overproduction of proinflammatory cytokines and tissue destruction [4].

Patients with NF suffer from pain, fever and erythema. Within a short period of time, the tissue gets tender, erythematous, overheated and blisters may develop. After some hours, a complete necrosis of the tissue is observed involving the subcutaneous tissue and underlying muscles. NF is quickly progressing and leads to severe, life threatening situations that require immediate antibiotic i.v. administration and surgical debridement of affect tissue even before the pathogen can be identified [5–7]. Delayed diagnosis of NF results in worsened outcome including death of the patient [8–10]. Mortality rates have been noted as high as 70% if left untreated.

Early diagnosis is missed or delayed in 85% to 100% of cases in large published series because of the lack of specific clinical features in the initial stage of the disease [11,12]. Clinically, the early phase of NF and cellulitis (erysipelas) due to infections with group A *Strepotococci* or *Staphylococci* can be very similar [5,9,11,13,14] and may lead to confusion and delay of treatment. Thus, the aim of the present study was to compare the initial clinical and laboratory presentation of NF and cellulitis in detail to assess if a typical pattern can be identified that aids early diagnosis of NF and avoidance of fatal outcome.

## Patients and Methods

### Patients

All patients with the diagnosis ‘necrotizing fasciitis’ (NF) that were admitted to the University Medical Center Mainz were identified using the patient administration software SAP searching for the ICD10 code M72.6. Between January 2001 and December 2010, 29 patients with this diagnosis were found. The NF patients were cared for in an interdisciplinary approach including intensive care.

To allow for a comparison to the clinical features of cellulitis (erysipelas), 59 age- and gender matched patients with this diagnosis (ICD 10 code A46.x) were identified using a similar approach. Since it is mainly the severe cases of erysipelas which are problematic with regard to the differential diagnosis NF, only those with a hospitalization time of ≥10 days were chosen.

The present retrospective study was approved by the local ethics committee, the ‘Ethik Kommission der Landesärztekammer Rheinland-Pfalz’. Any patient information was anonymized and de-identified before entry into the database and before analysis.

### Data assessment

All patient charts were subjected to a retrospective analysis of standardized parameters documenting 138 different variables including basic patient information (age, gender, body mass index, etc.), risk factors, prior co-morbidities, aetiology of current disease, localization, symptoms, diagnostical parameters, therapy and outcome (compare S1 Methods). The presence of comorbidities was documented as absent/present. Pain assessment was performed by categorization into ‘no pain/mild pain’ = 0, ‘intermediate pain’ = 1, and ‘strong pain’ = 2. Prior renal dysfunction was taken from the detailed patients’ history. Patients without prior renal disease that developed oliguria and/or elevated creatinine levels were graded as those with ‘acute renal injury’ as a result of the present condition.

### Statistical analyses

All information was entered into PASW Statistics 18.0. Descriptive Statistics was used to analyse the distribution of the variables. Comparisons between patient collectives were performed using the binary logistic regression. A p-value of ≤0.05 was considered significant.

## Results

The age of all 29 NF patients was 57.1±17.1 years (mean ±SD, range 20–91 years), amongst whom 18 (62%) were male and 11 (38%) female. The 2:1 matched patient collective with cellulitis (n = 59) consisted of 36 male (61%) and 23 (39%) female patients, mean age 57.9±17 years.

### Clinical presentation upon admittance

**Fig 1:**
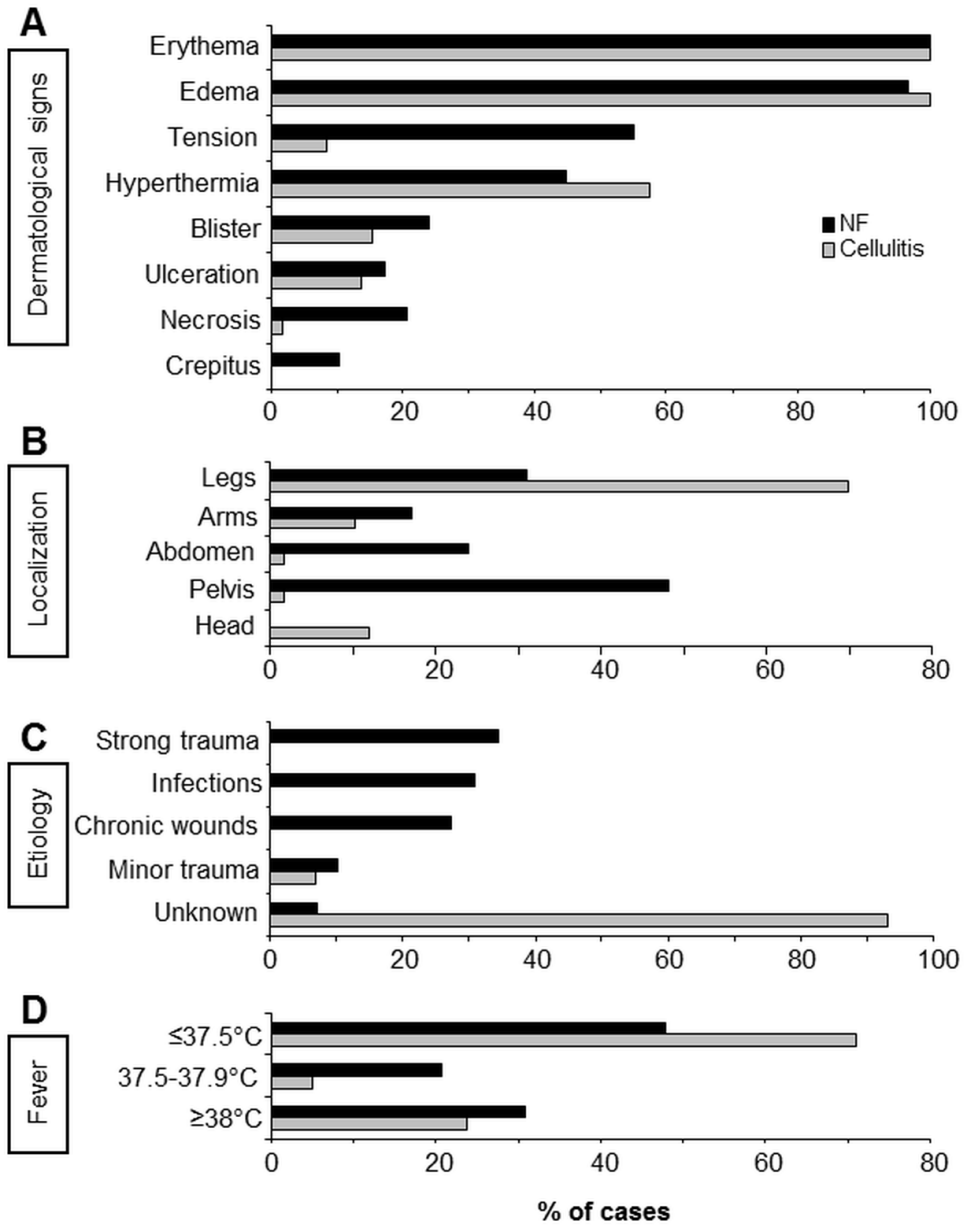
Clinical presentation of patients with necrotizing fasciitis (NF) as compared to cellulitis. All patient charts of 29 NF and 59 age- and gender matched patients with severe cellulitis were analysed retrospectively for various dermatological signs/descriptions of disease presentation, localization of disease, suspected etiology, and body temperature. All information is given in % of cases.

Upon dermatologic consultation, most skin lesions were described as erythematous and edematous; tenderness was observed mainly in NF, whereas slightly more cellulitis lesions were regarded as hyperthermic (Fig 1A). Secondary signs such as blister formation, ulceration, necrosis and crepitus were more often found in descriptions of initial presentation of NF as compared to erysipelas.

Disease localization of NF varied between the patients, whereas cellulitis patients showed involvement of primarily the lower legs in ~70% (Fig 1B). This is in line with the aetiology of NF development, since in most cases the reason for the infection was considered known (93.1%) (Fig 1C). Severe trauma (surgery) was seen as reason in 34.5% (10/29), prior infections in 30.9% (9/29, abscess n = 5, bursitis olecranii n = 2, tonsillitis n = 1, septic abortion n = 1), and chronic wounds in 27.5% (8/29, ulcerations n = 4, stool incontinence n = 2, diabetic ulceration n = 1, urostoma n = 1). Minor trauma (3/29) and unknown aetiology (2/29) were not found often in the NF patient files.

In contrast, despite of strong attempts to identify the reason for cellulitis development, in only 6.8% of cases the physicians documented minor traumas (4/59, minor trauma n = 1, insect bite n = 3) as portal of entry.

**Fig 2:**
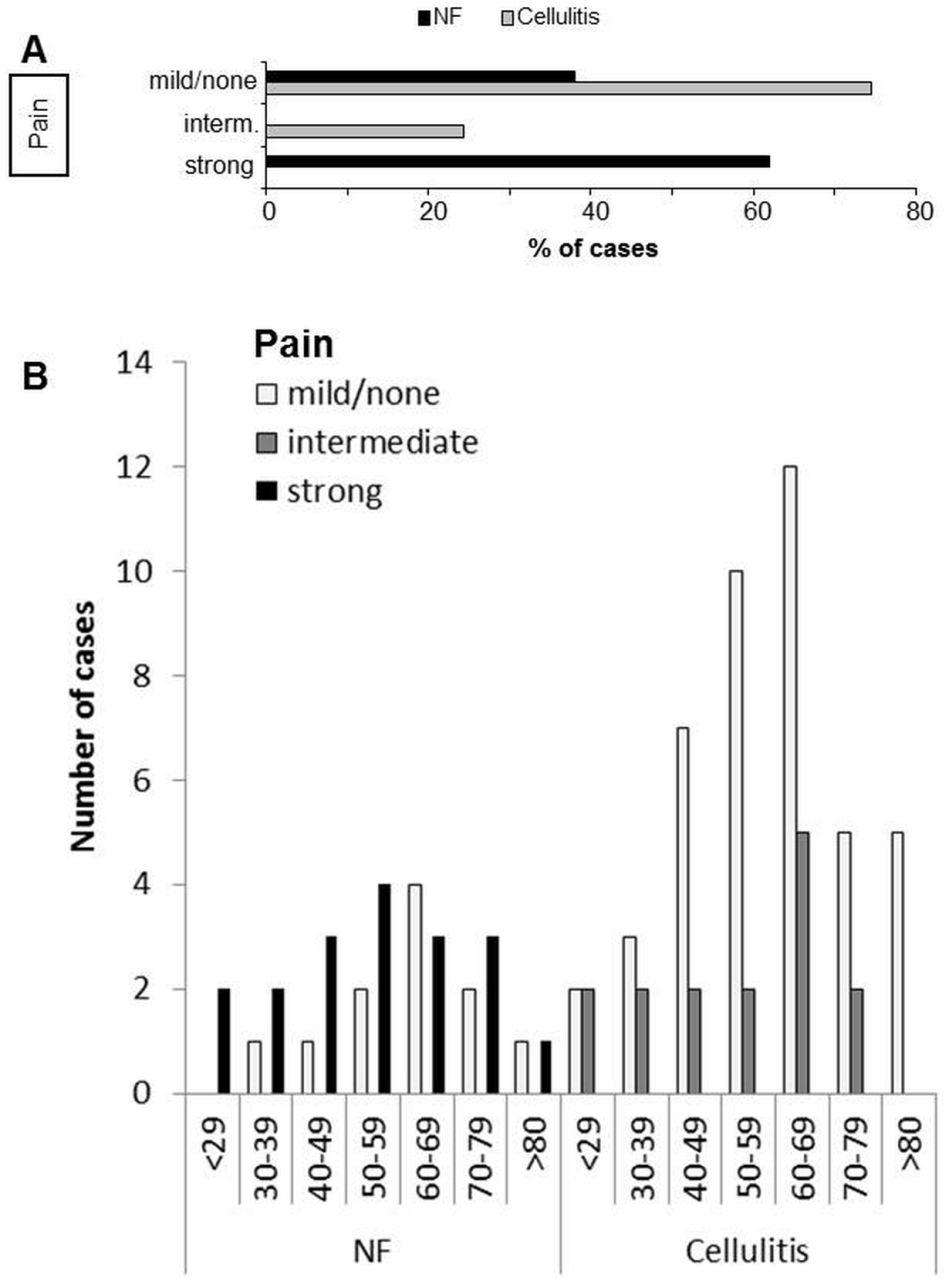
‘Pain out of proportion’ is indicative for necrotizing fasciitis (NF), but not cellulitis. Pain description in the patient charts of 29 NF and 59 age- and gender matched patients with severe cellulitis was scored as “mild/none”, “intermediate” or “strong”. A, al information is given in % of cases. B, Patients with NF or cellulitis were divided into the age groups indicated. Pain assessment for NF and cellulitis is depicted separately.

Both disease manifestations can be accompanied by elevated body temperature and painful lesions. Interestingly, a higher proportion of patients with NF showed signs of fever (15/29, 52%) as compared to patients with cellulitis (29%, 17/59, Fig 1D). Most striking, however, was the difference in the documented degree of pain (Fig 2A). NF patients complained about strong/very strong pain in 18/29 cases (62%), whereas only 14/59 (24%) of cellulitis patients indicated intermediate pain; strong pain was not documented in any case of cellulitis (0/59). In 38% (11/29) of NF and 75% (44/59) of cellulitis patients, respectively, pain was not documented in the files. Age-dependent differences in pain documentation were not obvious (Fig 2B).

The majority of NF patients also showed one or more signs of systemic disease (n = 22, 76%). Developing sepsis (n = 20), tachycardia (heart frequency >100 beats/minute; n = 11) and acute kidney injury (n = 10) were most common, but 5 patients were also disoriented and 4 showed multi organ failure. Other signs were disseminated intravascular coagulation (n = 2), nausea/vomiting (n = 1), and diarrhea (n = 1). Apart from chills in 14/59 cellulitis patients (24%), this patient collective did not show additional signs of systemic organ involvement. Interestingly, chills were not documented in any patient with NF.

In summary, the differences in clinical presentation of skin involvement in NF as compared to cellulitis were not dramatic and highlight the difficulties in differentiating NF from cellulitis clinically in an early stage [15]. In most cases, NF resulted from larger trauma as compared to cellulitis leading to a more broad distribution of affected body sites. Interestingly, however, NF patients suffered significantly more often from strong/very strong pain (‘pain out of proportion’), elevated body temperature and other early signs of systemic involvement.

### Co-morbidities

**Fig 3:**
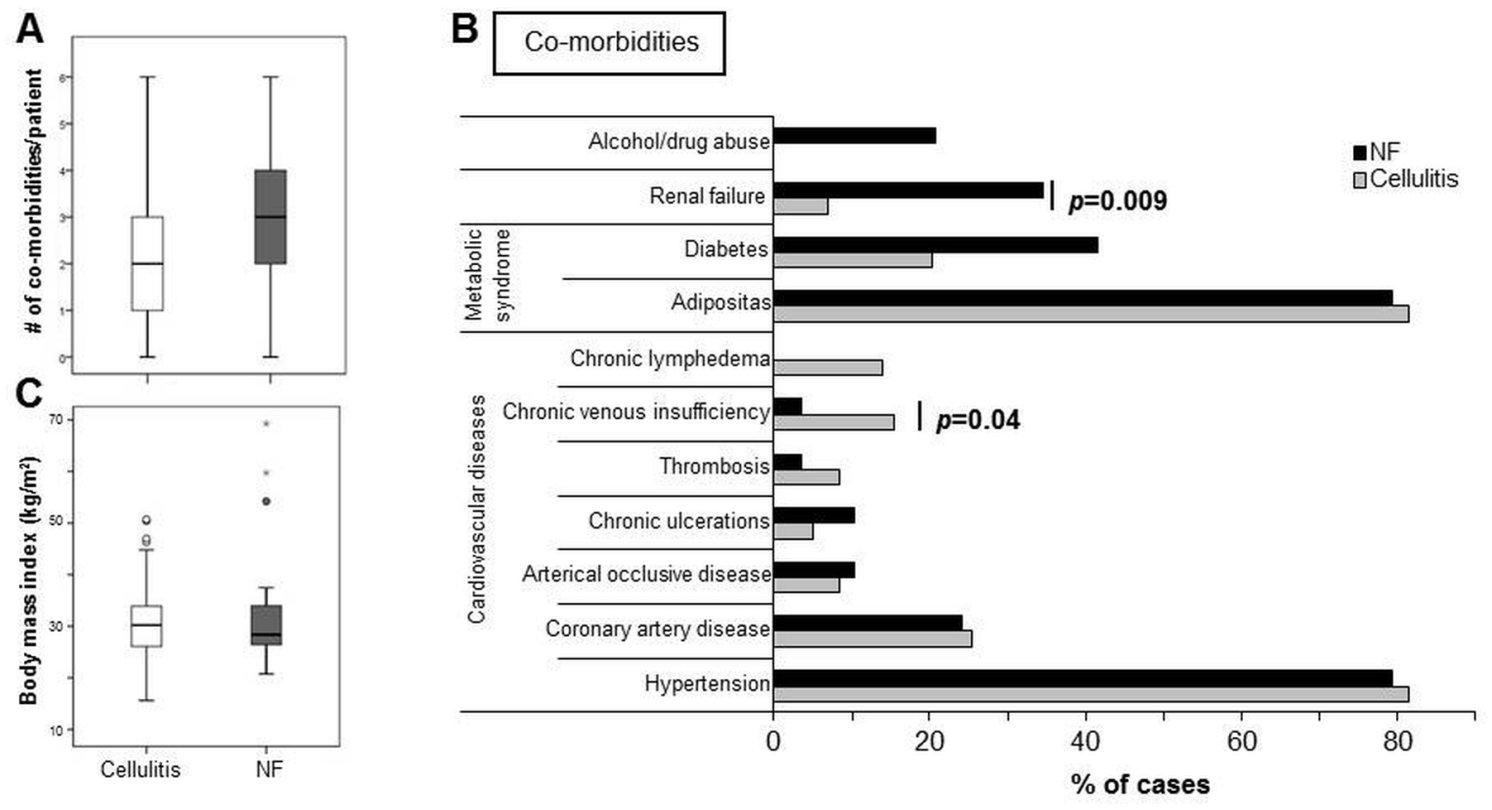
Assessment of co-morbidities in NF and cellulitis patients. A, The number of concomitant diseases was assessed for each case of NF (n = 29) and cellulitis (n = 59). B, For NF and cellulitis patients, the % of cases showing co-morbidities for each of the indicated diseases is shown. Statistical analyses using the binary logistic regression test revealed statistically significant differences for renal failure and chronic venous insufficiency. C, The body mass index was calculated for each patient. A+C, all data is shown as box plot.

Both patient collectives were characterized by additional morbidities as expected, but NF patients had slightly higher numbers of additional conditions as compared to cellulitis patients (2.9±1.7 vs. 2.3±1.6, respectively, Fig 3A). As indicated in Fig 3B and 3C, hypertension and obesity were frequent in both patient collectives. However, prior renal dysfunction (*p* = 0.009), diabetes mellitus type II and alcohol/drug abuse (*p* = 0.034) were (significantly) more frequent in NF patients in line with a previous study [16–18]. Interestingly, chronic venous insufficiency (*p* = 0.04), chronic lymphatic edema, and venous thrombosis were more frequently documented in patients who developed cellulitis.

### Laboratory findings

**Fig 4:**
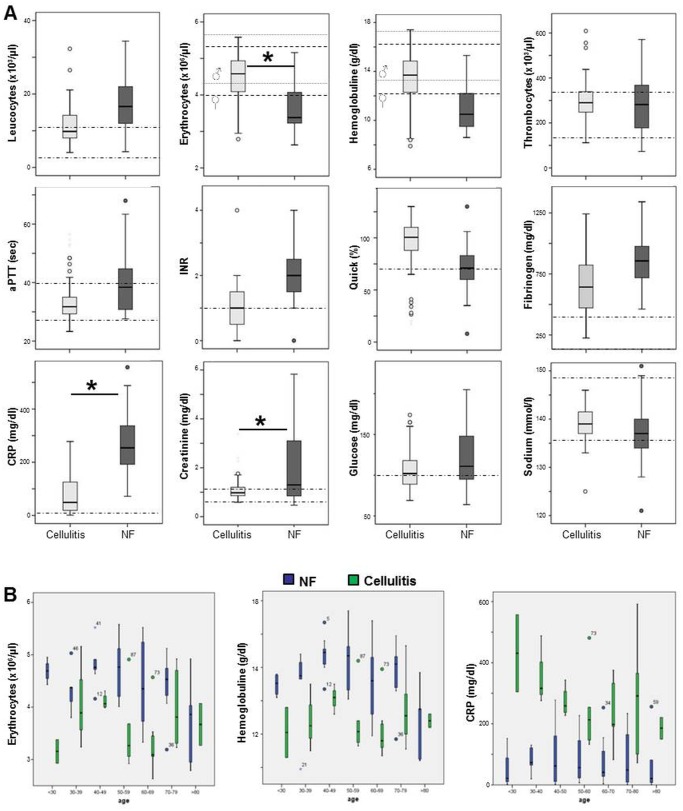
Laboratory findings in NF patients revealed significant signs of inflammation, renal failure and anaemia. Laboratory investigations were performed in most cases of NF and erysipelas. A, All findings were analysed using PASW Statistics 18.0 (IBM SPSS Inc.) and the binary logistic regression test; data are shown as box plots. Normal values/ranges are depicted as dotted lines (for erythrocyte count, and hemoglobuline gender specific ranges are given). Statistical differences between NF and cellulitis patients are indicated (* = *p*≤0.05). B, Laboratory values for erythrocyte counts, hemoglobulin and CRP were split by age groups as indicated.

We next studied the routine laboratory findings obtained upon admission of the patients. Normal levels were found in the number of thrombocytes, sodium, and glucose levels. All other parameters were pathologically altered as compared to healthy controls in both patient collectives, indicating systemic inflammation/infection (Fig 4).

In NF patients, elevated levels for the leukocyte count and fibrinogen were observed as compared to cellulitis. Most importantly, a strong difference in the CRP levels was observed between the two conditions: Patients with cellulitis exhibited an increased CRP of 49 mg/dl (median, range 1 to 278) which was well above normal values of healthy controls. NF patients, however, displayed ~5-fold higher CRP levels with 254 mg/dl (median, range 72 to 592). This difference was statistically significant (*p*<0.001).

Interestingly, in NF patients, the blood creatinine level was significantly increased (*p* = 0.01), which may indicated pre-existing renal dysfunction or ongoing early signs of acute kidney injury due to sepsis. In addition, both aPTT and INR were increased in NF patients compared to cellulitis, together with a decrease in erythrocytes and hemoglobulin, all of which may additionally indicate sepsis development and disseminated intravascular coagulation [19]

### Laboratory risk indicator for necrotizing fasciitis-score

In 2004, Wong *et al*. created a laboratory risk indicator for necrotizing fasciitis (LRINEC) score that can be utilized to risk stratify patients presenting with signs of cellulitis to determine the likelihood of NF being present [20]. It uses six different serologic parameters: C-reactive protein (CRP, >150 mg/l – 4 points), total white cell count (<15 x10^6^/mm^3^ - 0 points, 15–25 - 1 point, >25 - 2 points), haemoglobin (>13.5 g/dl – 0 points, 11–13.5 - 1 point, <11 - 2 points), sodium (<135 mmol/L - 2 points), creatinine (>141 μmol/L - 2 points) and glucose (>10 mmol/L – 1 point). A score of 6 or higher indicates that NF should be seriously considered. The LRINEC score, developed based on data from 89 NF patients compared with 314 severe cellulitis patients or abscess or both, is widely used, but has never been validated and the authors themselves noted that many other conditions might cause similar laboratory derangements [21].

**Fig 5:**
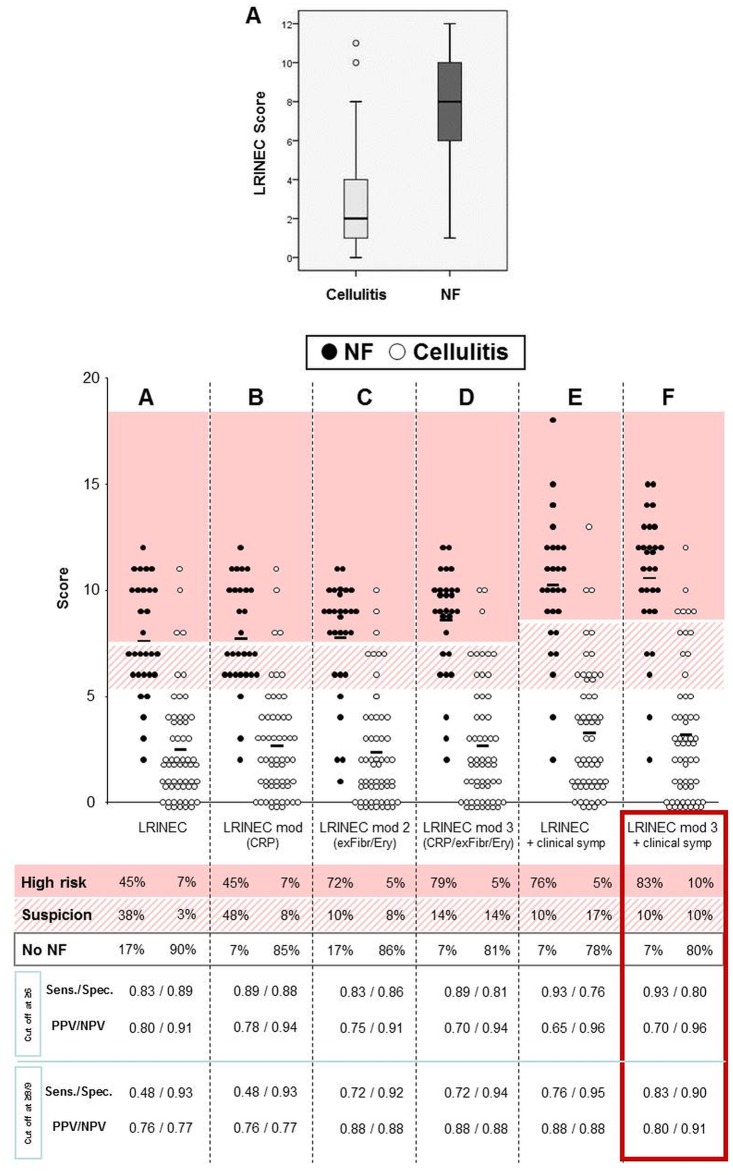
Modifications of the laboratory risk indicator for necrotizing fasciitis (LRINEC) strongly improves its clinical value. A, For a all 29 NF and 59 matched cellulitis patients, the LRINEC score was calculated. Results are shown as box plots. B-F, To improve its clinical relevance, several variations to the LRINEC sore have been introduced. In B, CRP levels were modified to 2 points (≥100 mg/dl) and 4 points (≥150). In C, sodium and glucose were exchanged for erythrocyte count (<4 x10^6^/μl– 1 point) and fibrinogen levels (>750 mg/dl– 2 points). In D, alterations of B+C were combined. In E, clinically, immediately obvious parameters were added as follows: pain (strong– 2 points, intermediate– 1 point, mild/none– 0 points), fever (≥38°C– 2 points, 37.6–37.9°C– 1 point, ≤37.5°C– 0 points), tachycardia (>100 heart beats/minute– 1 point), and signs of renal failure (– 1 point). F, combination of D and E. For all variations, the scores were ranked as ‘no signs of NF’ (white), ‘suspicious’ (stripes), and ‘clear signs of NF’ (pink). At the bottom, sensitivity (sens.), specificity (spec.), positive and negative predictive values (PPV/NPV) of the (modified) scores are shown.

Unlike in other studies [22–23], in our patient collective, the overall LRINEC score was significantly higher in NF patients as compared to the cellulitis collective (median 8 vs. 2, p>0.001) confirming the clinical relevance of this score (Fig 5A) [24,25]. In the NF patients, 45% had a score ≥8, 38% between 6–7, and 17% ≤5. In the matched cellulitis collective, only 7% had a score ≥8, 3% between 6–7, and 90% ≤5 (Fig 5B).

Importantly, with regard to the relevance of each of the parameters chosen for the LRINEC score, our study indicated that the CRP values are important and the levels of serum sodium and glucose are of less value. Thus, we assessed the benefit of different variations in the LRINEC score. First, a CRP >100 mg/l received 2 points, and >150 mg/l received 4 points (second panel from left, Fig 5B). This alteration mainly reduced the number of negative predictions. Next, we suggested to exchange glucose and sodium levels for erythrocyte count (<4 x10^6^/μl– 1 point) and fibrinogen levels (>750 mg/dl– 2 points). As a result, a significant improvement of the positive prediction value to 72% was noted (with additional 10% suspicious NF cases), without increasing the rate of false positive estimations of cellulitis cases.

Finally, adding relevant clinical parameters to the score such as pain (severe– 2 points, intermediate– 1 point, mild/none– 0 points), fever (≥38°C– 2 points, 37.6–37.9°C– 1 point, ≤37.5°C– 0 points), tachycardia (>100 heart beats/minute– 1 point), and signs of acute kidney injury (– 1 point) further optimized the score. Our cases of NF were now rated as 83% ‘strong suspicion’, 10% ‘suspicion’, and 7% ‘no signs of NF’. In the matched cellulitis collective, 10% were classified as ‘strongly suspicious’ for NF, 10% ‘suspicious’, and 80% showed no evidence. As a result, the positive predictive value (PPV) for cases with ‘strong suspicion’ was improved from 0.76 to 0.8, together with an improvement of the negative predictive value (NPV) from 0.77 to 0.91. Overall, the new modified LRINEC score also considering important clinical symptoms strongly increased the positive and reduced the negative predictive values of the score.

## Discussion

NF patients need to be identified quickly to avoid fatal outcome of this fulminant infection resulting in widespread necrosis of the affected tissue. Patients are often taken to surgery based on a high index of suspicion, determined by the patient’s signs and symptoms together with certain laboratory alterations. Especially in the early phase, other conditions such as cellulitis may present with similar features leading to misdiagnosis and delay of treatment.

A differentiation of cellulitis and NF may be possible in many cases via pathogen involvement. In our collective, 45% of the patients suffered from mono-microbial NF (of which 54% were Streptococci (with 46% and 8% group A and group B Streptococci, respectively), 46% Staphylococci among which 38% were *S*. *aureus* and 8% coagulase-negative Staphylococci including *S*. *lugdunensis*), whereas 48% of cultures revealed multiple organisms. In 7%, the pathogen was not identified. This is in line with prior findings about the frequency of mono- and polymicrobial NF [4,16,26]. Both conditions require early medical treatment consisting of antibiotics which should be started as soon as possible. Microbiological cultures are taken to determine the pathogens responsible, but only serve to ensure appropriate modifications in antibiotic treatment, since their results usually arrive too late to wait for before intervention. Initial treatment of NF (and cellulitis) often includes a combination of i.v. antibiotics including penicillin, and clindamycin.

In contrast to cellulitis and other related infections, aggressive surgical debridement (removal of affected tissue) is always necessary in NF patients to keep it from spreading and is the only treatment available [6]. Repeated explorations usually need to be done to remove additional necrotic tissue. Typically, this leaves large open wounds, which often require skin grafting. The associated systemic inflammatory response is usually profound and many patients require monitoring in an intensive care unit.

Laboratory findings may indicate the extent of infection. In line with prior findings, we confirmed that the LRINEC is helpful to identify patients at risk for NF as compared to severe cellulitis [16,17,27,28]. In our hands, differences in the levels of CRP were most prominent with a factor of x5 in NF. Additionally, elevated creatinine together with decreased hemoglobulin and erythrocyte counts are strong indicators for NF. Importantly, with regard to the relevance of each of the parameters chosen for the LRINEC score, our study demonstrated that the levels of serum sodium and glucose are of less value, whereas a modified score additionally considering erythrocyte count and fibrinogen levels is significantly more helpful. Futures studies may want to additionally consider other parameters, such as serum lactate [29].

Interestingly, our study revealed that clinical features show typical characteristics for NF patients. As expected, the early dermatologic description of NF and cellulitis did not differ. However, disease localization of NF more frequently involved body sites other than the lower extremities (which was most frequent in cellulitis) and trauma was often remembered. In addition, NF patients more frequently suffered from co-morbidities. Most importantly, however, the NF patients showed signs of systemic inflammation (fever, tachycardia, renal failure) and complained about strong/very strong pain. In our investigation, this “pain out-of-proportion” was one of the most prominent clinical features that helped to identify NF patients. Using these clinical parameters in combination with a modified LRINEC score (including fibrinogen levels and erythrocyte counts, Table 1), we showed that the positive predictive value of the score can be increased to 83% (strong suspicion) plus 10% (suspicion) compared to only 7% false negative predictions. Overall, however, the performance of the LRINEC mod 3 compared to LRINEC mod 3 + clinical symptoms was also improved as compared to the original score. Whether addition of pain assessment to the modified score is required will need to be assessed.

**Table 1:** Modified LRINEC score with clinical symptoms.

**Laboratory parameters**		
C-reactive protein (CRP)	>150 mg/dl	4 points
Total white cell count (WBC)	<15 x10^6^/mm^3^	0 point
	15–25 x10^6^/mm^3^	1 point
	>25 x10^6^/mm^3^	2 points
Erythrocyte count	<4 x10^6^/μl	1 point
Haemoglobin	>13.5 g/dl	0 point
	11–13.5 g/dl	1 point
	<11 g/dl	2 points
Creatinine	<135 mmol/L	2 points
Fibrinogen levels	>750 mg/dl	2 points
**Clinical parameters**		
Pain	mild/none	0 point
	intermediate	1 point
	strong	2 points
Fever	≤37.5°C	0 point
	37.6–37.9°C	1 point
	≥38°C	2 points
Tachycardia	>100 heart beats/minute	1 point
Signs of acute renal injury	no	0 points
	yes	1 point
**Sum:**		

Score results: ≥8: strong suspicion for NF; 6–7 suspicion¸ ≤5 no suspicion.

## Conclusions

In summary, in the present study we intended to stratify risk factors that allow for a better differentiation of NF and cellulitis at initial presentation to allow for quick recognition of NF. Several studies have previously demonstrated that early surgical debridement leads to significantly better survival rates and less post-NF morbidity [6,21]. Here, we report several clinical features to be risk indicators, among which the degree of pain and other signs of systemic involvement were most important. These findings together with a significantly elevated CRP of ≥150 mg/l as well as decreased hemoglobin and erythrocyte count would have led to the identification of 83% of the NF patients as compared to cellulitis (10% showed this risk pattern). The strong suspicion should prompt us to reinvestigate these patients frequently, since the overall dynamics of NF is rapid and within several hours severe disease progression will become obvious.

One limitation or the present study is the retrospective nature of the study. Utilization of the modified LRINEC in a prospective setting may not be as impressive due to unmeasured effects. In addition, only a limited number of cases with NF were available. The fact that the patients with NF had a significantly higher incidence of organ failure speaks to the problems in obtaining matched controls in a case-control study. A prospective trial of patients presenting with severe cellulitis compared to NF is needed.

Overall, we suggest implementing this new, modified LRINEC score into clinical daily routine to validate its relevance. In the long run, these measures may allow for an optimization of the care of NF patients leading to more rapid initiation of surgery and improved disease outcome.

## Supporting Information

S1 Methods Questionnaire for standardized assessment of relevant clinical and laboratory findings in patients with necrotizing fasciitis compared to severe cellulitis. (DOCX)
