# Supplementary material for: Improvement of a Clinical Score for Necrotizing Fasciitis: ‘Pain Out of Proportion’ and High CRP Levels Aid the Diagnosis
Source: PLoS One. 2015 Jul 21;10(7):e0132775. doi: 10.1371/journal.pone.0132775 (PMC4511009; doi:10.1371/journal.pone.0132775)
Supplement: S1 Methods — (DOCX) [file pone.0132775.s001.docx]

**Questionnaire/**

**Standardized Patient Assessment form**

General patient information:

 Name

 NF/ cellulitis

 Date of birth

 gender

 age at diagnosis

 height

 weight

Prior risk factors (co-morbidities):

 BMI

 Hypertonia

 Alcohol abuse

 HIV infection

 i.v. drug abuse

 Disorientation

 Thrombosis

 chronic venous insufficiency

 chronic lymph edema

 peripheral arterial obstructive disease

 coronary heart disease

 chronic ulcerations

 diabetes mellitus

 chronic renal dysfunction

 other:____________________________________

Possible etiological factors for current situation:

 Insect bite

 Injections

 Tonsillitis

 recent surgery at similar body sites

 minimal trauma

 Bursitis olecrani

 Syringe abscess

 Gravitation abscess

 Acute ulceration

 Diabetic ulceration

 Urostoma

 septic late abort

 Stool incontinence

 unknown cause

 other:____________________________________

**Symptoms**

Localisation:

 Abdomen

 Lower belly

 Genital area

 Perianal region

 right leg  left leg

 right foot  left foot

 right arm  left arm

 Face/neck

Clinical features:

 Pain (severe=2, medium=1, none=0)

 Disoriented appearance

 Temperature day before surgery, day 1, day 2, day 3

 Chills

 Tachycardia

 Multi organ failure

 Sepsis

 Acute kidney injury

 Disseminated intravasal coagulation

 Diarrhea

 Nausea/Vomiting

Skin characteristic:

 Ulcerations

 Erythema

 Edema

 Tenderness

 Hyperthermia

 Blisters

 Tissue necrosis

 Crepitus

**Diagnostic parameters:**

Laboratory values:

 CRP before surgery, day 1, day 2, day 3

 Leukocytes before surgery, day 1, day 2, day 3

 Sodium level

 Creatinine level

 Glucose

 Erythrocyte numbers

 Hemoglobin

 Thrombocyte numbers

 Activated Partial Thromplastin Time (aPTT)

 Quick

 INR

 Fibrinogen

Pathogens found in wound

 Name:_________________________

 Name:_________________________

 Name:_________________________

Radiology:

 X-rays

 Sonography

 MRT

 CT

**Therapeutic options chosen/Outcome:**

Surgical therapy

 Debridement

 Numbers of surgery

 Time interval until surgery

Antibiotics used:

 Name: _____________________________

 Name: _____________________________

 Name: _____________________________

Wound treatment:

 Vacuum assisted closure therapy (VAC)

 Hyperbaric oxygen therapy

 Split skin

Outcome:

 Time of hospitalisation

 Exitus letalis

 Amputation
